# A Graph-Based Author Name Disambiguation Method and Analysis via Information Theory

**DOI:** 10.3390/e22040416

**Published:** 2020-04-07

**Authors:** Yingying Ma, Youlong Wu, Chengqiang Lu

**Affiliations:** 1 School of Information Science and Technology, ShanghaiTech University, Shanghai 201210, China; mayy1@shanghaitech.edu.cn; 2 Shanghai Institute of Microsystem and Information Technology, Chinese Academy of Sciences, Shanghai 200050, China; 3 University of Chinese Academy of Sciences, Beijing 100049, China; 4 University of Science and Technology of China, Heifei 230026, China; lunar@mail.ustc.edu.cn

**Keywords:** name disambiguation, graph neural network, clustering analysis, mutual information

## Abstract

Name ambiguity, due to the fact that many people share an identical name, often deteriorates the performance of information integration, document retrieval and web search. In academic data analysis, author name ambiguity usually decreases the analysis performance. To solve this problem, an author name disambiguation task is designed to divide documents related to an author name reference into several parts and each part is associated with a real-life person. Existing methods usually use either attributes of documents or relationships between documents and co-authors. However, methods of feature extraction using attributes cause inflexibility of models while solutions based on relationship graph network ignore the information contained in the features. In this paper, we propose a novel name disambiguation model based on representation learning which incorporates attributes and relationships. Experiments on a public real dataset demonstrate the effectiveness of our model and experimental results demonstrate that our solution is superior to several state-of-the-art graph-based methods. We also increase the interpretability of our method through information theory and show that the analysis could be helpful for model selection and training progress.

## 1. Introduction

Entity Linking tasks recognize or disambiguate named entities to an entity in a knowledge base. It is a significant problem in natural language processing and has been extensively studied. One important task of entity linking is author name disambiguation. The author name disambiguation task aims to partition publications written by different people who share the same name such that each partition only contains documents associated with one real-life person. In the field of bibliographic data analysis and document retrieval, author name disambiguation is crucial. For instance, when someone is looking for the publications of a scholar name “Charles” in a database, the query may return many papers from different “Charles”, which could cause ambiguity and deteriorate the performance of this search. The efficiency of the search could be significantly enhanced if the search results were distinguished by name references. Besides, if an organization want to calculate the impact of many authors, they need to know their publications exactly.

There are two types of questions that could be answered by solving author name disambiguation task. One is to find out who writes the specific paper and another is to decide which articles are written by this author. Author name disambiguation becomes more challenged when dealing with scholars not from western countries, as people with different names share the same spelling. For example, there are more than 300 thousand people named “Wei Zhang” in China.

Despite the fact that the problem has been studied for decades, it remains largely unsolved. Some researchers consider author name disambiguation as a classification task and manage to predict the correct label for each paper [1] or predict whether two documents are related to the same author [2]. A large number of labels are needed in classification tasks. Other works focus on clustering methods, which are usually unsupervised but lead to relatively poor performance. Graph networks have attracted more and more attention in recent years. As co-authors and their publications could naturally construct author-author network and document-document network, the author name disambiguation task could be solved by incorporating graph neural networks.

Handling name ambiguity problems presents several challenges:

- **Should we use labeled data?** The disambiguation performance of supervised methods is generally better than unsupervised methods because of utilizing labeled data. But the size of datasets is usually large and manually labeling all tags will consume a lot of manpower and time. Solving this problem by semi-supervised methods has become a more practical solution to this problem.
- **How do we make the best of information and present a comprehensive model that could fit most datasets?** Most existing methods are usually based on either feature extraction or on paper relationship and author relationship. Feature extraction methods adopt a large number of attributes and formulate many rules to measure the similarity between documents. When some attributes are missing, the rules will be invalidated. Research based on relationship graphs ignores some basic attributes of documents and reduces the performance of name disambiguation. It is necessary to design a basic method to deal with all kinds of datasets with different attributes.
- **Does the proposed method work well on larger datasets?** Many state-of-art methods dealing with author name disambiguation are evaluated on relatively small document sets, associated with no more than twenty author name references. When the dataset becomes larger, the performance may drop a lot.
- **Why does the proposed method work well?** Graph-based name disambiguation approaches can be interpreted as introducing relationship. We wish to know what role the training process plays and why some approaches lacking interpretability perform well.

In this paper, we propose a name disambiguation method that could generate document embedding vectors without labeled data and utilizes the true number of clusters for clustering.

Our method could make the best of the attributes of documents and the relationship of papers as well as the relationship of co-authors. Incorporating a word2vec model, we use basic attributes for feature extraction: title, author, organization and construct document representation vectors. The word2vec model could work on the dataset with some missing data and is flexible to involve other features. Two models are used for representation learning afterward. A Graph Auto-Encoder (GAE) is applied to construct a paper-paper network based on feature similarity and a Graph Embedding model is used to utilize the graph topology information of co-author relationship. Our method dealing with basic attributes could be easily applied to other author datasets.

A larger dataset sampled from AMiner [3] is used for evaluating the disambiguation results of our model. Experimental results indicate that our solution achieves significantly better performance than several state-of-the-art graph-based methods including Zhang and Yao [4], Zhang et al. [5] and GHOST [6]. Our method finds the document embedding vectors based on an unsupervised learning method and is even superior to the semi-supervised method [4].

Besides, we try to increase the interpretability of our method through information theory. Through the analysis of changes in mutual information, we could learn more about our model and the training progress.

In addition, visualization through dimensionality reduction is used for comparing our approach and other’s work and assessing the role of each component.

The remainder of this paper is organized as follows: In Section 2, we introduce recent related works on name disambiguation, including the supervised, unsupervised, semi-supervised and graph-based methods, and works on the information plane. In Section 3, we formalize the problem with preliminaries. The framework is presented in Section 4 and the calculation of mutual information is posed in Section 5. We evaluate our method in Section 6. In Section 7, we conclude the article.

## 2. Related Work

### 2.1. Name Disambiguation

A variety of methods have been proposed to solve the issue of author name ambiguity. Some researchers treat it as a classification task, predicting the correct labels of each paper or determining whether two articles are written by the same author. Classification tasks require lots of labels, so such tasks are usually supervised.

Wang et al. [1] proposed a boosted tree classification method for determining true papers and false papers of a set of homonymous authors in academic research processes. The input of the models is the attributes of publications, including title, author, affiliation, abstract, etc. A deep neural network model to learn attributes automatically is used in [2]. Some researchers have noticed the potentials of graph-based feature and utilized a Bi-directional Long Short-Term Memory to encode graph attributes for classification [7]. Many traditional methods measure similarity metrics by cosine similarity of term frequency-inverse document frequency (TD-IDF). Others train a model to learn a vector representation by triplets samples without considering any structure information. Kim et al. [8] proposed a hybrid model that makes use of both and train an SVM, a RF, a GBT and a DNN to determine whether a pair of publications is related to the same author or not.

Some works make use of external data collected from the web. A disambiguation Support Vector Machines (SVM) kernel is trained to leverage the rich structure and extensive coverage of the information encoded in Wikipedia in [9]. Han et al. [10] offered a generative Naive Bayes probability model and an SVM model. They illustrate these two approaches on data collected from the web and DBLP citation database (https://dblp.uni-trier.de).

In real digital libraries, the cost of human annotation for each name is unaffordable and supervised classification solutions are inefficient and inappropriate. Therefore, some unsupervised clustering models are applied to this task. For unsupervised name disambiguation, publications are divided into several clusters in order that each one only contains records associated with one person.

Some papers studied the efficiency of different clustering algorithms, such as Adaptive Threshold Clustering (ATC) [11] and Bayesian Information Criterion-mean (BIC-mean) [12]. Cen [13] modeled a new pairwise similarity measure by optimizing the linear regression model and then adaptively cluster documents by optimizing a regression function. A unified probabilistic framework and a hierarchical agglomerative clustering method based on Dempster–Shafer theory (DST) [14] propose new similarity metrics in name disambiguation task. They embed each document in a low dimensional vector space for clustering. Some probabilistic relational [15,16,17] models are also unsupervised.

The main drawback of supervised methods is that a large amount of labeled data requiring expensive human annotation is needed while unsupervised clustering methods usually perform worse than supervised solutions. The following methods are based on semi-supervised algorithms.

Levin et al. [18] proposed a two-stage method that combines the classification and clustering method. In stage one, they find positive document pairs that are probably written by the same researcher through high-precision rules, while articles that are not linked by the rules are negative ones. In stage two, the positive and negative document pairs are taken as training data for a supervised classifier that predicts whether two articles are related to the same author or not. Predictions of the classifier are then used as a similarity metric for clustering. Louppe et al. [19] posed phonetic-based blocking strategies for pre-clustering, a linkage function to learn an accurate classifier for identifying authors and semi-supervised agglomerative clustering of signatures. Blocking strategies are applied for placing documents that are likely to be written by the same author in the same area.

Some graph-based approaches are also used in this task due to the growing research interests in graph neural networks. Unsupervised learning approach using K-way spectral clustering [20] could divide a graph into several parts for clustering. Ref. [21] constructs a co-authors graph and applies graph structural clustering method ’gSkeletonClu’ to detect outliers and identify overlapping communities. GHOST [6] utilizes a coauthorship graph to compute the similarity between node pairs, utilizing the attribute of coauthor only could achieve the same performance as previous complicated approach. A combined graph encompassing the author-author graph and document-document graph is put forward by Pooja [22]. Each connected component of the combined graph represents a distinct cluster. In [23], researchers designed a deepwalk algorithm on the author-author graph and document-document graph and embedded graph nodes such that the document representations contain the information of the graph topologies. A pairwise factor graph (ADANA) [24] and title-coauthor graph (GFAD) [25] are also proposed.

In previous work [4], global metric learning and local linkage graph are combined. This method learns low-dimensional documents embedding vectors from their attribute characteristics. In global metric learning, the authors sampled real positive and negative pairs for triplet learning and utilized Graph Auto-Encoder in local graph learning. However, they did not consider the relationship between authors and used a lot of labeled samples. Zhang et al. [5] pre-processed the input data as three graphs: person-person graph, document-document graph, and person-document graph. They projected the data into low dimensional space. However, features other than co-author relationship are not taken into account and a lot of information is lost. Our proposed method takes advantages of the above two methods by leveraging both attributes and relationships.

Apart from author name disambiguation, some researchers are devoted to person name disambiguation in web search and the solutions are very similar to author name disambiguation. In [26], web pages are clustered after feature selection and measuring similarity as well.

### 2.2. Information Plane

The amount of information between a hidden layer and input/output can then be measured over training progress, yielding a picture of the optimization process in the information plane [27]. Works via information plane gives us a better understanding of neural networks.

Tishby [28] visualized the mutual information of hidden layers regarding input and output of a fully connected neural network via information plane and provided a better understanding of Deep Neural Networks (DNN) and the optimization process. They suggested that the goal of the network is to optimize the Information Bottleneck tradeoff between compression and prediction. The training progress could be divided into two phases by visualization. In phase one, hidden layers keep extracting information from input variables (I(X;T) increases during phase one while *T* represents random variables of hidden layers). In phase two, the layers remove irrelevant information (I(X;T) decreases).

Cheng et al. [29] verified the validation of information plane on classic Convolutional Neural Network including AlexNet and VGG. They learn the capability of these CNN structures based on the information plane.

On the one hand, we wish to know whether our graph networks still follows the process of extracting information and compression in the IB principle. On the other hand, the variation of mutual information I(X;Y) could reflect information flow from a variable *X* to a variable *Y*. We calculate the mutual information of latent vectors regarding input and output to find out whether IB principle holds and increase the interpretability of our framework.

## 3. Preliminaries

### 3.1. Problem Formulation

In this section, we formalize the name disambiguation problem. Firstly, we introduce the notation used in this paper.

Let D be the set of all documents in the database, and |D| is the number of the documents. For an author name *i*, $D^{i}=\left\{d_{1}^{i},d_{2}^{i},\ldots ,d_{N}^{i}\right\}$ is a set of |N| documents associated with the name. Each document has a set of attributes, like coauthor, title, venue, affiliation, keywords, etc. We let $d_{n}$ be the *n*-th document, $d_{n}$ could be represented as $\left\{f_{1},f_{2},\ldots ,f_{M}\right\}$, in which *f* is a stemmed word of each attribute and *M* stands for the number of stemmed words of these attributes. Let A be the collaborator set, then it can be expressed as $A=\{a_{1},a_{2},\ldots ,a_{L}\}$ with *L* co-authors. The notations used in this article are summarized in Table 1.

In the name disambiguation task, *i* often represents an author name instead of a real-life person. This means $d_{1}^{i}$ and $d_{2}^{i}$ may not be written by the same author. Our task is to find the correct partition function such that each partition only contains documents related to one person *p*. Given a dataset $D^{i}$, we hope to group the documents into *K* disjoint clusters $C^{i}=\left\{C_{1}^{i},C_{2}^{i},\ldots ,C_{K}^{i}\right\}$ where the number of clusters is unique for each author *i*. *K* is the number of real people that share the same author name. The partition function is:

$$\Phi \left(D^{i}\right)\to C^{i}$$

where $C_{k}^{i}=\left\{d_{j}^{i}\right|\Phi \left(d_{j}^{i}\right)=p_{k},d_{j}^{i}\in D^{i}$}, denoting the paper cluster of a real-life person $p_{k}$, k∈{1,2,…,K}.

### 3.2. Mutual Information and Information Bottleneck

Given random variables (*X*, *Y*), mutual information I(X;Y) is a measurement of the mutual dependence between the two variables calculating by their marginal probability distributions p(X), p(Y) and joint distribution p(X,Y):

$$I\left(X;Y\right)=\sum_{x,y}p\left(x,y\right)\log\frac{p(x,y)}{p(x)p(y)}$$

The entropy of variable *X*, denoted by H(X), standing for uncertainty for random variables is defined as:

$$H\left(X\right)=-\sum_{x}p\left(x\right)\log p\left(x\right)=I\left(X;X\right)$$

Let x∈X be the inputs of a neural network and y∈Y be corresponding labels, t∈T are the output of hidden layers. In general neural networks, Y→X→T forms a Markov chain. Information Bottleneck principle is proposed to provide a technique for extracting information from the input random variables, which is related to predicting some output random variables. In other words, Representation *T* is a compression of *X* and a prediction of *Y*. In learning progress, neural network models need to learn an efficient representation *T* of *X* while preserve prediction capability of *Y*, which could be formulated by the following optimization function:

$$\min_{p(t|x),Y\to X\to T}\left\{I\left(X;T\right)-\beta I\left(T;Y\right)\right\}$$

where β is a tradeoff parameter between compression and prediction accuracy. In DNNs and CNNs, IB principle claims that deep neural networks consist of two stages of fitting and compression.

## 4. Framework

In this section, we present details of our name disambiguation model. We first introduce document representation and variational Graph Auto-Encoder, which mainly focus on encoding attributes of each paper. The similarity metric is calculated by the relationship between these attributes. Then we introduce Graph Embedding to our model to leverage the relationships between authors.

**Figure 1:**
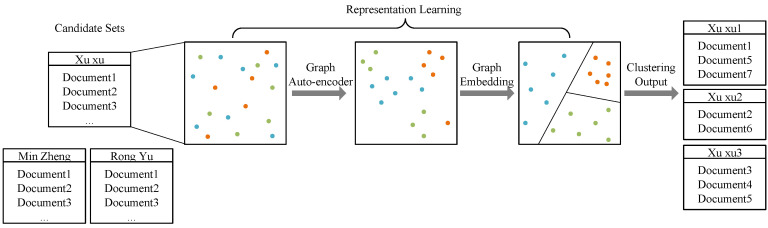
This figure shows the framework of our model. After constructing representation vectors by a word2vec model, we do representation learning through a Graph Auto-Encoder and a Graph Embedding model. Then we cluster the documents using their representation vectors.

As shown in Figure 1, the colorful dots represent document representations. We use a word2vec model to calculate these representation vectors. Then a variational Graph Auto-Encoder and a Graph Embedding model are applied to fine-tune these vectors. After representation learning, we use Hierarchical Agglomerative Clustering to partition documents into different clusters.

### 4.1. Feature Embedding

Recent works to learn the representation of words mainly focus on neural network-based methods. Among these works, word2vec models [30] including Continuous Bag-of-Words (CBOW) and Skip-gram model could extract the relationships between words and perform better than other works.

Given a series of training words $w_{1},w_{2},\ldots ,w_{T}$, CBOW learns word representation based on the co-occurrence of words within the size of a predefined context window in the training corpus. The model is based on the idea that the probability of occurrence of words around a word can predict what the word is. It maximizes the co-occurrence probability between words that appear in the predefined context window. The objective is to maximize the probability:

$$\frac{1}{T}\sum_{t=1}^{T}\sum_{-c\leq j\leq c,j\neq 0}\log p\left(w_{t}|w_{t+j}\right)$$

in which *c* is the context window and the loss function is optimized by a neural network. After training, the weight matrix is used to represent the training words.

A specific document $d_{n}$ could be represented as $\left\{f_{1},f_{2},\ldots ,f_{M}\right\}$ and *f* denotes the stemmed words of the title, co-authors, orgnization and venue. One hot vector is utilized to stands for each feature *f*. We use CBOW model here to compress each document representation into a low-dimensional vector. After training the neural network, the *m*-th weight ${\hat{f}}_{m}$ of the neural network weight matrix is the low-dimensional representation related to feature $f_{m}$. Every document representation $d_{n}$ could be calculated by $d_{n}=\sum_{i=1}^{M}\alpha_{i}{\hat{f}}_{i}$. $\alpha_{i}$ is the inverted document frequency of $f_{i}$ to reduce the weight of some irrelevant stemmed words like prepositions. $D^{i}=\left\{d_{1},d_{2}\ldots ,d_{N}\right\}$ captures the similarity between these documents using the co-occurrence probability of each paper’s features.

### 4.2. Variational Graph Auto-Encoder

Recall $D^{i}$ is the set of document representation related to the author name *i*. We remove the superscript *i* in the notations for simplicity and the implication of the symbol is unchanged. For each author name, we could build a graph G=(D,E) where $D=\{d_{1},d_{2}\ldots ,d_{n}\}$ is the document representation output $D^{i}$ defined in Section 4.1. The document embedding vectors could represent graph nodes and E is the adjacency matrix *A* to represent edges between these nodes.

The adjacency matrix *A* is constructed by two approaches: one is calculating affiliation similarity between two documents, and the other one is using a collection of high-precision features.

We measure the similarity between two papers by the weighted sum of feature representation. If the similarity between document $d_{i}$ and $d_{j}$ is larger than the threshold, we construct an edge between these two nodes and $A_{ij}=A_{ji}=1$. We empirically set this threshold to 25. For other node pairs, the relevant value in the adjacency matrix is 0. Due to the lack of labeled data, we also use some high precision rules to bootstrap positive pairs. For example, there is little chance that two authors with the same name work in the same organization and have co-authors work together. Besides, one’s corporators are relatively fixed in a period of time. If two publications are written by exactly the same co-authors, an edge is also constructed between them.

**Figure 2:**
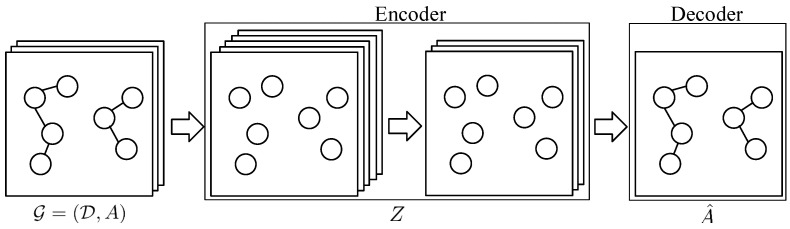
Variational Graph Auto-Encoder.

Variational Graph Auto-Encoder (as shown in Figure 2) is used in our model to improve the generalization ability of the model. Let $X=[d_{1}^{T},d_{2}^{T},\ldots ]$ be the representation matrix of documents associated with an author name. The encoder is a two-layer graph convolutional network:

$$q\left(Z|X,A\right)=\prod_{i=1}^{N}q\left(z_{i}|X,A\right)$$

with

$$q\left(z_{i}|X,A\right)=N\left(z_{i}|\mu_{i},\mathrm{diag}\left(\sigma_{i}^{2}\right)\right)$$

$\mu =\left[\mu_{1},\mu_{2},\ldots \right]=GCN_{\mu }\left(X,A\right)$ is mean value matrix and $\log\sigma =\left[\log\sigma_{1},\log\sigma_{2},\ldots \right]=GCN_{\sigma }\left(X,A\right)$ represents variance matrix. The two-layer GCN is computed by

$$GCN\left(X,A\right)=\tilde{A}Relu\left(\tilde{A}XW_{0}\right)W_{1}$$

where $W_{0}$ is identical in $GCN_{\mu }\left(X,A\right)$ and $GCN_{\sigma }\left(X,A\right)$ while $W_{1}$ is different, $\tilde{A}=D^{-\frac{1}{2}}AD^{\frac{1}{2}}$ and *D* is degree matrix. The decoder reconstructs the adjacency matrix $\hat{A}$:

$$p\left(A|Z\right)=\prod_{i=1}^{N}\prod_{j=1}^{N}p\left(A_{ij}|z_{i},z_{j}\right)$$

with

$$p\left(A_{ij}|z_{i},z_{j}\right)=\mathrm{sigmoid}\left(z_{i}^{T}z_{j}\right)$$

The goal is to make the reconstruction matrix as close as possible to the original one. The loss function is as follows:

$$L=E_{q(Z|X,A)}\left[\log p\left(A|Z\right)\right]-\mathrm{KL}\left[q\left(Z|X,A\right)||p\left(Z\right)\right]$$

The first item refers to cross entropy loss. In the second item, $p\left(Z\right)=\prod p\left(z_{i}\right)$, $p\left(z_{i}\right)=N\left(0,1\right)$ is standard normal distribution. KL[q(·)||p(·)] is the Kullback-Leibler divergence which denotes the similarity between distribution *p* and *q*.

We take the latent matrix output of encoder $Z=[z_{1}^{T},z_{2}^{T},\ldots ]$ as new embedding vectors of these documents. By knowing the relationship of existing nodes, the new matrix is a higher-level expression and have the predictability of new node pairs.

### 4.3. Graph Embedding

In the Graph Auto-Encoder, we only consider the relationship of co-authors and affiliations between two documents. As demonstrated in Table 2, we cannot determine whether document 1 and document 2 are written by the same author “Yanjun Zhang” by measuring feature similarity. If we have a look at document 3, we could know that one-hop collaborators of document 1 and document 2 are completely overlapped, so the “Yanjun Zhang” in document 1 and document 2 is the same real-life person. In order to utilize relationships of co-authors and papers, the intuition is to represent a graph network as a matrix.

There are several ways to extract graph topology information, like deepwalk [31] and graph convolutional network [32]. Inspired by previous work [5], we try to make related authors and papers closer utilizing author-author, author-document, and document-document graphs. Different from their model, we wish to initialize the author and document node representation with prior knowledge. We preserve the ranking orders based on letting the embedding vector of a document closer to all related items (including documents and their authors).

Given an author name *i*, we construct a person-person network $G_{pp}=\left(A,E_{pp}\right)$ refering to the collaborators’ relationship within all documents related to author *i*. A is the set of all co-authors related to author name *i* and $e_{ij}\in E_{pp}$ is the edge between author $a_{i}$ and author $a_{j}$. The weight of edge $e_{ij}$ is the number of collaborations between two authors and the value is 0 if they never co-published a paper.

Similarly, a document-document network $G_{dd}=\left(D,E_{dd}\right)$ reflects the graph topology between the set of documents associated with author *i*. $E_{dd}$ considers one-hop co-authors. If two documents have more than one one-hop collaborator, they are likely to be similar and we build an edge between them. D is constructed by the output matrix *Z* of Graph Auto-Encoder such that we include feature engineering into Graph Embedding.

More importantly, we would like to introduce author relationship to guide the document clustering. $G_{pd}=\left(A\cup D,E_{pd}\right)$ is the bipartite network that connect documents and authors. $A^{i}$ is the set of collaborators of all documents in $D^{i}$. The edge is defined as the number of author $a_{i}$ appears in the document $d_{j}$ and the value is 1 if the paper is written by this author and otherwise 0. If two papers have a large overlap of co-authors set (their authors are close to each other in the author-author graph topology), they are considered to be similar as well.

The similarity score between two documents is defined as $S_{ij}^{dd}=d_{i}^{T}d_{j}$. Let document *i* and document *j* be positive pairs, we would like to maximize the $S_{ij}=d_{i}^{T}d_{j}$ such that the cosine similarity is smaller. We find node pairs based on the ranking orders and the probability of preserving the orders is defined as:

$$p\left(S_{ij}>S_{it}|d_{i},d_{j},d_{t}\right)=\mathrm{sigmoid}\left(S_{ij}^{dd}-S_{it}^{dd}\right)$$

For a set of documents D, the probability is:

p(D)=∏(di,dj)∈PGdd(di,dt)∈NGddsigmoid(Sijdd−Sitdd)

Aiming to fine-tune the document embedding vectors, we need to maximize the probability. The loss function of the linked document network is:

$$L_{dd}=\min_{D}-\ln p\left(D\right)$$

In the same way, we could derive the loss function for preserving the ranking orders of the author-author graph and bipartite network:

$$L_{pp}=\min_{A}-\ln p\left(A\right)$$

$$L_{pd}=\min_{A,D}-\ln p\left(A,D\right)$$

For this three Graph Embedding model, our goal is to embed these three graphs, so the optimization function is:

$$L=\min_{A,D}L_{dd}+L_{pp}+L_{pd}+\lambda (||A^{\prime }{\left|\right|}_{F}^{2}+{\left|Z|\right|}_{F}^{2})$$

where $A^{\prime }$ and *Z* are co-author matrix and document matrix.

After training the Graph Embedding model, we combine the topology information of three graphs as well as feature similarity into the document embedding matrix. Let the output embedding matrix be $Y=[y_{1}^{T},y_{2}^{T},\ldots ]$ and for every author name the matrix is different.

### 4.4. Clustering

We apply Hierarchical Agglomerative Clustering (HAC) [33] afterward. This algorithm treats each data point in the training sample set as a cluster at first. Then it calculates the distance between every two groups and merges the two most similar clusters that are closest until the number of clusters equal to the number of true clusters.

## 5. Calculating Mutual Information

Researchers plot information plane by calculating mutual information I(X;T) and I(T;Y). The calculation of mutual information in classification tasks have been studied. In this section, we will show how we do calculations step by step in a clustering problem.

As our proposed method consists of several parts, we evaluate I(X;T) and I(T;Y) separately for each component. In our task, input *X* stands for the original representation matrix. The original document representation is calculated by the CBOW model. Therefore, $X=D=\{d_{1},d_{2},\ldots ,d_{N}\}$. And *Y* stands for true clustering labels.

Tishby [28] utilized toy data for demonstration and separated the neuron’s output into 30 bins between −1 and 1. In practical deep neural networks with real datasets, *T* is usually a high-dimensional matrix. Thus, in our calculation, a softmax function is applied to the output of each component for squashing a C-dimensional vector *z* to a C-dimensional vector with values in the range [0,1]:

$$\mathrm{sigmoid}{\left(t\right)}_{j}=\frac{e^{t_{j}}}{\sum_{i=1}^{C}e^{t_{i}}}$$

Now we get output of neurons ranging from 0 to 1 and then we also bin each neuron’s output into 30 bins range from 0 to 1. The output of this discretization progress is representation *T*.

Cheng [29] noted that the network’s parameters Θ are fixed after training, which means $p(t_{i}|x_{i})=1$. $t_{i}$ could be represented as $t_{i}=f\left(x_{i},\Theta \right)$. In this case, H(T|X)=0. The mutual information between *X* and *T* could be calculated by:

$$I\left(X;T\right)=H\left(T\right)-H\left(T|X\right)=H\left(T\right)=-\sum_{t}p\left(t\right)\log p\left(t\right)$$

Representation *T* contains information about input *X* and thus the calculation takes this information into account. During our calculation, the network’s parameters are also fixed. Therefore, we use the above formula to calculate I(X;T). In Feature Embedding, representation *T* is exactly *X* and mutual information can be calculated by I(X;T)=I(X;X)=H(X).

Different from image classification tasks, embedding matrix *T* of documents is not directly associated with labels in our model. We use HAC to establish links between documents. Therefore, I(T;Y) is estimated by the mutual information between the output of HAC and labels. Thus, in our approach I(X;T) represents the relation between the outputs of each component and original document embedding vectors. I(T;Y) is another measurement of clustering results.

## 6. Experiments

### 6.1. Datasets

Many existing name disambiguation methods are validated by no more than 20 author datasets. We evaluate our proposed approach on a dataset of online academic search and data mining system AMiner [3]. This system automatically extracts and storages researchers’ personal files from the web. It also integrates academic data from other online databases such as DBLP, ACM Digital Library, CiteSeer, and SCI. For a fair comparison, we validate our approach with the same sampled dataset used in [4] consisting of 100 author names. Some methods require external information (e.g., social linkage) that is hard to access and not provided by popular open datasets. Therefore, these models are not compared in our experiments.

### 6.2. Competing Method

We validate the performance of our model by comparing it with several state-of-the-art graph-based models as following:

**Zhang and Yao [4]:** The method proposes a name disambiguation method by incorporating both global and local information. In global supervision, human annotation is required to generate triplet sampling, while in the local context, a Graph Auto-Encoder is used.

**Zhang et al. [5]:** This method constructs three local graphs within an author name according to author and paper similarity. By optimizing the pairwise ranking objective, the graph topology is embedded into a low-dimensional matrix.

**GHOST [6]:** GHOST is a model based on author acquaintance. Every author’s name associated with papers represents a node in the GHOST graph. By selecting valid paths, it computes the similarity between two nodes and uses the AP algorithm for clustering.

**Union finding:** Union finding is to build graph linkage based on several strictly matching of co-authors and affiliations. A clustering is constructed by all nodes that have a corporate relationship.

### 6.3. Experimental Results

We evaluate the results with pairwise precision, recall and F1-score and calculate macro score of each metric for all 100 names. For a fair comparison, we select the same 15 name references as in [4] and compare these three metrics for each name reference.

We use the same hyperparameters for each dataset. In the CBOW model, the dimension of document representation vectors is set to 100 and the context window is set to 5. In the variational Graph Auto-Encoder, the threshold of inverse document frequency is 25, the output dimension of the first layer in graph convolution network is 200, the output dimension of the second layer is set to 100, the learning rate is 0.01 and the model trained for 200 epochs. In the graph network embedding model, the learning rate is 0.05 and the regularization parameter is 0.01.

Table 3 shows that the average F1-score of our methods is +3.87% higher than Zhang and Yao, +25% over Zhang et al., and +33.85% over GHOST. It performs the best for 11 (out of 15) name references.

Our model achieves the best performance comparing with other graph-based methods in F1-score. Further, we would like to know whether changes in precision or recall lead to the variation in F1-measure. Comparing pairwise precision between our solution and other methods, we could see that the average precision of our approach is a little higher than that of Zhang and Yao, Zhang et al. and the Union finding, but lower than GHOST. Moreover, the average recall of our clustering results is much higher than the approach Zhang and Yao, Zhang et al. and GHOST, but lower than Union Finding. High recall value demonstrates that our model is capable to find more documents that belong to the right categories, thus the proportion of positive pairs in all true pairs increases. Although Union finding achieves the highest recall, this method would divide documents that do not belong to author *i* into the cluster of his documents and make the wrong predictions, leading to much lower precision. Similarly, GHOST is more conservative by ignoring many documents which belong to the author. Overall, our method performs the best.

GHOST constructs a similarity matrix according to the co-author graph. Zhang et al. leverage the graph topology by relying on a co-author graph and the paper-paper graph. Zhang and Yao utilizes global supervision and local linkage paper-paper graph. Some methods focus on the relationship between two documents, and others concentrate on graph topology among the set of all authors. By combining the advantages of both and making the best of the information, our model outperforms these baseline methods, even if we do not use labeled data as Zhang and Yao did to generate triplet samplings.

**Figure 3:**
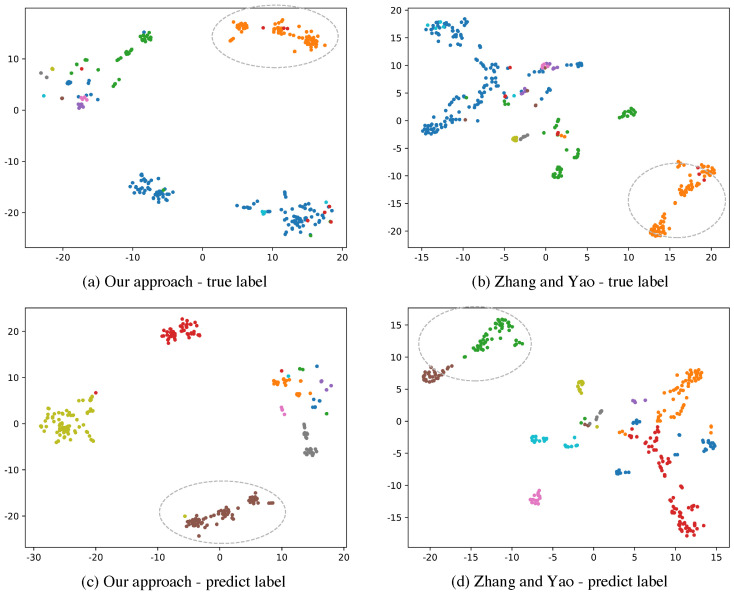
Visualization of clustering results of our proposed method and method of Zhang and Yao. We compress the representation vector space into a 2-dimensional space on a candidate set. Different colors in (**a**,**b**) represent different true clusters while different colors in (**c**,**d**) denote different predicted clusters.

Figure 3a,b are true cluster visualization of embedding spaces while Figure 3c,d denote low dimensional space of predict clusters. Different categories are distinguished by different colors. The F1-score of our approach on this set is 0.6338 while the method of Zhang and Yao only achieves 0.5382. From the circles with dashed lines in Figure 3b,d we could see that samples in a true cluster are clustered into different parts in prediction because of the spatial dissimilarity of representation vectors using their model. Our approach does better clustering by allowing similar vectors to be closer. The orange scattered points shown in Figure 3a indicate that these representation vectors are closer in the embedding space by our approach. These scattered points are classified correctly in Figure 3c.

### 6.4. Component Analysis

Each component in our approach is evaluated separately. Feature Embedding represents using only document representation to measure similarity. Graph Auto-Encoder and Graph Embedding are constructed on previous Feature Embedding. As shown in Table 4, compared with Feature Embedding, Graph Auto-Encoder and Graph Embedding improve the performance by 6.41% and 4.83% respectively. Our method is superior to the above sub-systems as the precision and recall are both the highest.

**Figure 4:**
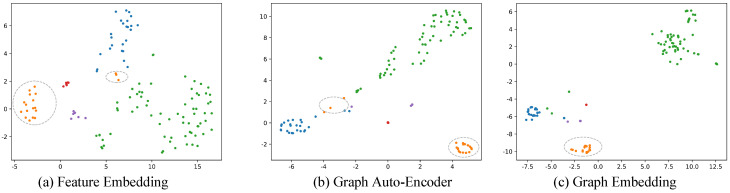
Visualization of clustering results of each component. Different colors denote different true clusters. Dashed grey ellipses indicate document representations in a true cluster are moved closer and closer by our method.

In addition, we evaluated each component by reducing the dimension of embedding vectors associated with a publication set. As shown in Figure 4, we use true labels distribution to reflect the impact of each sub-system on the embedding space. As we can see, Graph Auto-Encoder brings the green dots as well as the blue dots together while Graph Embedding lets these points be closer so that they can be clustered more accurately. Outlier orange dots, which are circled by grey dash lines, could also be found and moved to the correct area. This means that our model is effective for outliers.

### 6.5. Evaluating by Information Theory

We calculate I(X;T) and I(T;Y) on a candidate set for each component respectively and make more explanations of our model. *X* is the original document representation after feature embedding, *T* is the output of each component and *Y* stands for the matrix of true clustering labels.

From Table 5, we observe that I(X;T) is 5.3230 for Feature Embedding and after one epoch training of Graph Auto-Encoder the value drops to 3.8771, which suggests that introducing linkage function by Graph Auto-Encoder weakens the relationship between the new embedding matrix and the original one. Meanwhile I(T;Y) instantly increases to 2.2304. Also, we could see that the trends of I(T;Y) and F1-score are exactly the same. Both of them can reflect the quality of the clustering results.

Researchers found DNNs and CNNs could be divided into two phases of extracting information and compression via information plane. We wish to know whether our graph networks still follows this two-stage process. Although during experiments, our graph networks do not work this way. we could still utilize a similar method to find out what role each component plays and how it affects the final result.

**Figure 5:**
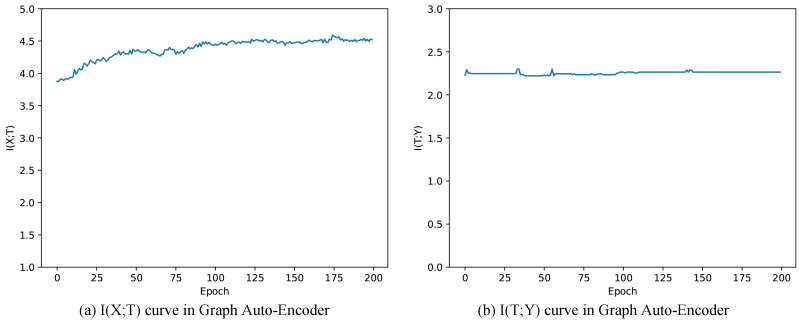
Mutual information paths of Graph Auto-Encoder.

Figure 5a,b are mutual information paths through training Graph Auto-Encoder. In Figure 5a, I(X;T) keeps increasing during the training progress, indicating that training Graph Auto-Encoder could learn more information from *X*. Meanwhile, mutual information I(T;Y) tends to be stable and increases a little (equals to 2.2781 after 200 epochs, see in Table 5). Combining with the fact that I(T;Y) instantly increases after one epoch training (shown in Table 5), we could infer that the improvement of clustering results is mainly based on the structure itself. As we introduce the linkage function of paper relationships with Graph Convolutional Network in GAE, other GCNs are worth trying for solving this problem. Besides, we could do less training without damaging the performance.

**Figure 6:**
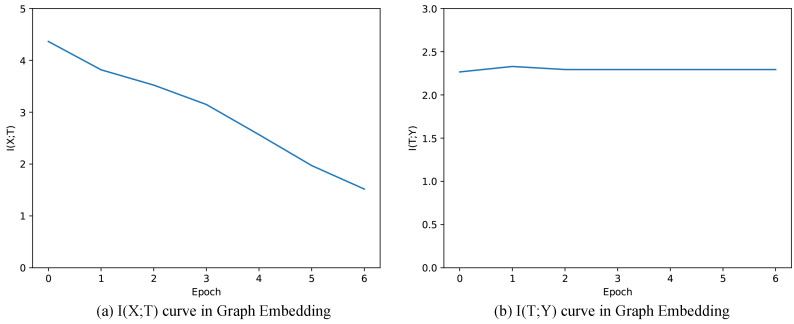
Mutual information paths of Graph Embedding.

Figure 6 shows the mutual information of I(X;T) and I(T;Y) when training Graph Embedding. We observe that I(X;T) keeps decreasing during training progress, which is contrary to the case in Graph Auto-Encoder. This is mainly due to the intrinsic property of the Graph Embedding that continuously introduces additional information in the process of training. In our experiment, Graph Embedding supplements *T* with additional information of person-person, document-document, person-document graph topology, leading to weaker and weaker dependence between *X* and *T*, and thus I(X;T) keeps decreasing during training. Similar to the scenario of Graph Auto-Encoder, I(T;Y) only slightly increases during the training progress (from 2.2659 to 2.2801, see in Table 5), coinciding with the small improvement of F1-score (from 0.7918 to 0.8155, see in Table 5). During the actual training progress, I(T;Y) and F1-score increases during the first several training epochs, but decreases after training too many rounds. The reason for this is that I(X;T) is too small, suggesting that we lose too much information from the original representation matrix. As the original representation vectors are constructed by attributes, there is a tradeoff between information of relationships and attributes. As we train these components separately, the model could not incorporate the information from both attributes and graph topology at the same time. Thus, one model incorporating relationships and attributes may be better for clustering.

In Information Bottleneck principle, I(X;T) decreases as hidden layers keep compressing information from input *X* while in our model the decrease suggests introducing additional information. Although the analysis results are not the same, but the analysis method utilizing mutual information is consistent and gives us a better understanding of these models.

## 7. Conclusions

In this paper, we introduce a method to address the problem of author name disambiguation. Our proposed representation model is capable of encoding attributes and graph topology of all authors and papers by incorporating a Graph Auto-Encoder and a Graph Embedding model. We evaluate our approach on a sampled dataset of an online academic search and data mining system which integrates data from popular databases. Experimental results show that our approach outperforms several state-of-the-art graph-based methods. In addition, we increase the interpretability of each component through information theory and show that it is capable of guiding the model selection and training progress in future work.

## Figures and Tables

**Table 1:** Notations.

Name	Description
D	the set of all documents included in the database
|D|	the number of documents in the database
*i*	author name *i*
$D^{i}$	the document set associated with the author name *i*
$d_{n}^{i}$	document *n* associated with the author name *i*
*f*	a stemmed word of an attribute
*p*	real-life person *p*
$A^{i}$	the set of all collaborators of author *i*
$C^{i}$	clusters for author *i*

**Table 2:** Related documents according to one-hop co-author.

Document 1: Author: **Yanjun Zhang**, Jun Shan, Zhanjun Liu, Fanqin Li Orgnization: College of Chemical Engineering, Department of Applied Chemistry,
Document 2: Author: **Yanjun Zhang**, Guanghan Zuo, Jun Ji Orgnization: College of Chemical Engineering Ctr. For Element and Strct. Anal.
Document 3: Author: Jun Shan, Zhanjun Liu, Guanghan Zuo, Jun Ji Orgnization: Department of Applied Chemistry, Ctr. For Element and Strct. Anal.

**Table 3:** Clustering Results of Different Name Disambiguation Methods. The F1-scores highlighted in bold show that our approach performs the best for 11 (out of 15) name references.

Name	Our Approach			Zhang and Yao			Zhang et al.			GHOST			Union Finding		
	Prec	Rec	F1	Prec	Rec	F1	Prec	Rec	F1	Prec	Rec	F1	Prec	Rec	F1
Xu Xu	70.73	70.06	**70.39**	74.18	45.86	56.68	47.73	39.98	43.51	61.34	21.79	32.15	7.22	66.29	13.02
Rong Yu	73.07	42.81	53.99	89.13	46.51	**61.12**	66.53	36.9	47.47	92.00	36.41	52.17	16.00	41.22	23.05
Yong Tian	78.12	61.03	**68.53**	76.32	51.95	61.82	73.18	56.34	63.66	86.94	54.58	67.06	10.78	94.50	19.36
Lu Han	60.02	35.29	**44.45**	51.78	28.05	36.39	46.05	17.95	25.83	69.72	17.39	27.84	15.10	96.30	26.10
Lin Huang	74.72	50.43	**60.21**	77.10	32.87	46.09	69.43	33.13	44.86	86.15	17.25	28.74	5.91	33.76	10.06
Kexin Xu	91.09	89.65	90.37	91.37	98.64	**94.87**	85.74	44.13	58.27	92.90	28.52	43.64	77.63	83.62	80.51
Wei Quan	77.23	48.86	**59.85**	53.88	39.02	45.26	74.41	33.94	46.62	86.42	27.80	42.07	37.16	96.57	53.67
Tao Deng	76.38	53.82	**63.15**	81.63	43.62	56.86	55.25	27.93	37.11	73.33	24.50	36.73	12.55	64.76	21.03
Hongbin Li	70.09	78.48	**74.05**	77.20	69.21	72.99	65.79	52.86	58.62	56.29	29.12	38.39	12.92	94.59	22.73
Hua Bai	65.86	41.94	**51.25**	71.49	39.73	51.08	54.93	35.97	43.47	83.06	29.54	43.58	22.08	93.23	35.71
Meiling Chen	79.60	47.84	**59.76**	74.93	44.70	55.99	79.22	25.15	38.18	86.11	23.85	37.35	24.83	66.92	36.22
Yanqing Wang	49.00	66.49	56.42	71.52	75.33	**73.37**	72.73	42.62	53.74	80.79	40.39	53.86	24.12	66.95	35.46
Xudong Zhang	74.55	22.24	**34.26**	62.40	22.54	33.12	55.63	8.11	14.16	85.75	7.23	13.34	65.12	47.36	54.84
Qiang Shi	53.38	50.36	**51.83**	52.20	36.15	42.72	43.33	37.99	40.49	53.72	26.80	35.76	18.11	86.37	29.94
Min Zheng	59.85	20.76	30.82	57.65	22.35	**32.21**	53.62	17.63	26.54	80.50	15.21	25.58	11.95	74.48	20.60
Average	78.10	67.47	**72.40**	77.96	63.03	69.70	70.22	48.72	57.53	81.72	40.43	54.09	40.78	76.52	53.20

**Table 4:** Clustering Results of Each Component.

	Prec	Rec	F1
Feature Embedding	72.29	50.14	59.21
Graphh Auto-Encoder	75.53	58.01	65.62
Graph Embedding	77.71	54.46	64.04
Overall	78.10	67.47	72.40

**Table 5:** Mutual Information and F1-score for Each Component.

	I(X;T)	I(T;Y)	F1
Feature Embedding	5.3230	2.0379	0.6209
Graph Auto-Encoder (after one epoch training)	3.8771	2.2304	0.7834
Graph Auto-Encoder (after training)	4.5186	2.2781	0.7939
Graph Embedding (after one epoch training)	4.3635	2.2659	0.7918
Graph Embedding (after training)	1.5175	2.2801	0.8155
